# Applicability of resting energy expenditure prediction equations in Chinese nursing home residents

**DOI:** 10.3389/fnut.2026.1860294

**Published:** 2026-07-28

**Authors:** Zhen Xu, Tianlun Li, Shuya Du, Mengjia Yue, Lu Wang, Jing Sun, Yanjuan Zhao, Xingyang Chen

**Affiliations:** 1 Department of Nutrition, Central China Fuwai Hospital, Central China Fuwai Hospital of Zhengzhou University, Zhengzhou, China; 2 Department of Nutrition and Food Hygiene, School of Public Health, Jilin University, Changchun, China; 3 Department of Clinical Nutrition, Zhengzhou University People’s Hospital, Henan Provincial People’s Hospital, Zhengzhou, China; 4 Department of Nutrition, The Third Affiliated Hospital of Zhejiang Chinese Medical University, Hangzhou, China

**Keywords:** indirect calorimetry, nursing home, older adults, predictive equation, resting energy expenditure

## Abstract

**Objective:**

This study aims to evaluate the applicability of 11 commonly used resting energy expenditure prediction equations among elderly residents in Chinese nursing homes and to investigate the key factors influencing their predictive performance.

**Methods:**

This study employed a cross-sectional design and included 156 elderly residents of nursing homes as study participants. The study conducted a comparative analysis of resting energy expenditure values measured using indirect calorimetry and the values predicted by 11 different prediction equations. In addition, the performance of each equation was comprehensively evaluated through comparisons of bias, precision, and accuracy, as well as Bland–Altman plots. Subgroup analysis and restricted cubic spline models were used to investigate the factors influencing the predictive performance of the equations.

**Results:**

A total of 156 older adults residing in nursing homes were included in the study, with a mean age of 83.4 ± 5.6 years; 62.2% were women. REE measured by indirect calorimetry was 1378.6 ± 268.0 kcal/d. Among the 11 predictive equations, only the Owen equation (1348.9 kcal/d, *p* = 0.113) and the Cunningham equation (1351.5 kcal/d, *p* = 0.155) showed no statistically significant difference between their predicted values and the measured REE; the remaining 9 equations all significantly underestimated REE (all *p* < 0.001). Among all equations, the Owen and Cunningham equations exhibited the lowest bias (0.2% and 0.7%), the best precision (150.7 kcal/d and 153.7 kcal/d), and the highest prediction accuracy (48.1% and 45.5%). However, Bland–Altman analysis showed that the 95% limits of agreement were wide (Owen: −401.2 to 460.6 kcal/d; Cunningham: −419.7 to 473.9 kcal/d), suggesting that prediction errors at the individual level remain substantial for both equations. Subgroup analyses revealed that age, muscle mass, and nutritional status were significantly associated with predictive performance; restricted cubic spline analysis further confirmed that age and skeletal muscle index exhibit nonlinear relationships with prediction errors.

**Conclusion:**

The Owen and Cunningham equations can serve as relatively preferred tools for estimating REE in older adults residing in Chinese long-term care facilities; however, their accuracy in predicting individual REE remains limited, and they are unable to fully account for the significant individual metabolic variations within this population. Overall, the application of existing REE prediction equations among older adults in Chinese nursing homes still has certain limitations. Therefore, when conditions permit, indirect calorimetry should remain the method of choice for assessing individual REE in older adults. Future large-sample, multicenter studies are needed to develop and validate REE prediction models better suited to the Chinese elderly population, thereby improving the accuracy of individualized energy requirement assessments.

## 1 Introduction

As the global population continues to age, nutritional health issues among the elderly have gradually become a key focus in the field of public health. In China, with the continuous improvement of the elderly care service system, an increasing number of older adults are choosing to reside in nursing homes, making the nutritional status of this population particularly worthy of attention. Previous studies have shown that the prevalence of malnutrition or the risk of malnutrition among elderly residents in Chinese nursing homes ranges from 31.37% to 55.6% (1). Malnutrition not only accelerates skeletal muscle loss and weakens immune function in the elderly but is also closely associated with increased hospitalization rates, rising medical costs, and an elevated risk of mortality (2).

Resting energy expenditure (REE) refers to the minimum energy required by the body to maintain basic vital functions—such as respiration, heart rate, and cellular metabolism—while awake, at rest, and in a fasted state; it accounts for approximately 60%–75% of total energy expenditure in humans (3). Due to declining physiological functions, older adults in long-term care facilities generally have lower levels of daily physical activity, and their basal metabolic rate gradually decreases with age, resulting in an overall lower REE. When an older adult’s energy intake does not match their actual REE requirements—whether due to insufficient or excessive intake—it may lead to malnutrition or obesity and their associated complications, thereby further exacerbating health impairments in older adults (4). Therefore, accurately measuring and assessing REE in older adults is of great significance for improving their health status in long-term care facilities.

Currently, indirect calorimetry is widely regarded as the gold standard for measuring REE. This method accurately measures oxygen consumption and carbon dioxide production during rest and calculates energy expenditure using relevant equations, yielding accurate results (5). However, this method requires high-quality equipment and highly skilled operators, and instruments are expensive and involve complex procedures. Its routine application in primary care settings such as nursing homes faces numerous limitations (6), making it difficult to meet the screening and assessment needs of large-scale elderly populations.

Therefore, in practical clinical nutritional assessment, clinicians typically rely on various REE prediction equations to estimate the REE of target populations. Among these, the Harris–Benedict, Mifflin–St Jeor, Owen, and Cunningham equations are the most widely used. It should be noted that the original samples for these commonly used prediction equations primarily come from European and American populations (7), whereas different ethnic groups exhibit significant differences in body composition and metabolic characteristics. Existing studies have confirmed that, at the same body mass index (BMI), the body fat percentage of Asian populations is generally higher than that of European and American populations (8). These differences in body composition may affect the applicability of REE prediction equations developed for European and American populations in Asian populations, leading to biased prediction results.

Relevant systematic reviews indicate that traditional prediction equations such as the Harris–Benedict equation have an accuracy rate of only 40%–60% for estimating REE in healthy older adults (9), demonstrating limited accuracy. Furthermore, in the specific population of older adults residing in long-term care facilities, although some studies have suggested that factors such as muscle mass, nutritional status, and inflammation levels may affect the applicability of REE prediction equations (10), systematic subgroup analyses targeting this population remain insufficient, resulting in a significant evidence gap.

This evidence gap may, to some extent, limit the accuracy of clinical nutritional assessments, leading to energy supply plans based on REE estimates from prediction equations that do not align with the actual needs of older adults, thereby affecting the efficacy of nutritional interventions. Based on this, this study adopted a cross-sectional design, using REE measured by indirect calorimetry as the gold standard, to systematically evaluate the predictive performance of 11 commonly used REE prediction equations in older adults residing in Chinese nursing homes. Concurrently, subgroup analyses were conducted to further explore the potential influence of factors such as gender, age, BMI, skeletal muscle index (SMI), calf circumference (CC), nutritional risk, and lifestyle on the performance of the prediction equations. This study aims to provide more targeted guidance for assessing energy requirements in elderly residents of nursing homes, thereby promoting the development of clinical nutrition management toward greater precision and individualization. It also seeks to contribute supplementary evidence to research on REE prediction in elderly Asian populations and enrich the body of research data in this field.

## 2 Methods

### 2.1 Study design and participants

This study is a cross-sectional study conducted at a 300-bed nursing home. The study population consists of elderly individuals who received routine care between November 2023 and December 2023. The inclusion criteria are: (1) Age ≥ 60 years; (2) voluntarily signed an informed consent form and completed all study procedures. The exclusion criteria included: (1) the presence of diseases or conditions affecting metabolism, such as pulmonary disease, fever, rheumatoid arthritis, or thyroid dysfunction; (2) the use of medications known to affect resting energy expenditure, including anesthetics and benzodiazepine sedatives; (3) receipt of enteral nutritional support; (4) Failure to complete resting energy expenditure measurements or body composition analysis. This study was approved by the Medical Ethics Committee of Henan Provincial People’s Hospital (<2023 > No.156). The final study participants were all older adults without significant physical disabilities, known diseases affecting energy metabolism, or mental disorders.

### 2.2 Indirect calorimetry

Resting Energy Expenditure was determined using indirect calorimetry, with measurements taken by technicians using a respiratory indirect calorimeter (ExpressCCM, Medgraphics, USA). Subjects were required to fast for at least 8 h the night before the measurement and avoid moderate- to vigorous-intensity physical activity. On the day of the measurement, upon arrival at the testing room, subjects rested in a supine position for at least 15 min in a quiet, isolated environment maintained at a temperature of 23 °C–25 °C. The technician then placed the subject’s head inside a transparent hood for measurement in accordance with the instrument’s operating procedures. The initial 5 min of data were used for adaptation to the equipment and environment and were excluded from the final analysis. Steady state was deemed to have been reached when the variability in both VO₂ and VCO₂ was less than 10% for 5 consecutive minutes, after steady state was achieved, technicians continued to collect gas exchange data for 30 min (11). If speaking, changes in body position, coughing, sleep, or other circumstances causing significant fluctuations in gas exchange occurred during the measurement, data from the corresponding time periods were excluded from the analysis. The final REE calculation was based on the average of data from continuous, stable time periods after steady state was achieved and that met quality control requirements. Resting energy expenditure is calculated using the simplified Weir equation based on the measured oxygen consumption (VO₂) and carbon dioxide production (VCO₂): REE = (3.94 × VO₂ + 1.11 × VCO₂) × 1,440 (12). All data measurements were performed by professionally trained technicians from the Department of Nutrition at Henan Provincial People’s Hospital and Central China Fuwai Hospital of Zhengzhou University in accordance with standardized procedures. Throughout the entire process, the subjects were closely monitored by the technicians to ensure they remained awake, calm, and at rest. Before each measurement, the indirect calorimeter was adequately warmed up and calibrated in accordance with the manufacturer’s instructions, and formal measurements were initiated only after successful calibration. During data collection, the complete measurement system was validated weekly using an ethanol combustion test. Instrument performance was considered acceptable only when the relative errors between the measured and stoichiometrically predicted values of both VO₂ and VCO₂ were ≤5% (13). Participant measurements were performed only after these acceptance criteria were met. The relative error for ethanol combustion validation was calculated as: Relative Error (%) = ∣X_measured_ − X_theoretical_∣/X_theoretical_ × 100%, X represents either VO₂ or VCO₂, and the theoretical values were derived from the stoichiometric equation of ethanol combustion. Within 1 week after completion of the formal study, three independent performance verification sessions were conducted. A total of 20% of the participants were randomly selected for repeat REE assessment, with a different subset included in each verification session. Repeat measurements were performed using the same CCM Express indirect calorimeter, measurement configuration, environmental conditions, and standardized operating procedures as those used during the original study. Relative error was calculated as: Relative error(%) = ∣REE_repeat_ − REE_original_∣/REE_original_ × 100%, Measurement stability across the three verification sessions was assessed using the coefficient of variation, and a relative error of ≤5% was prespecified as indicating acceptable measurement agreement (14).

### 2.3 Body composition analysis

Body composition analysis was performed using multi-frequency bioelectrical impedance analysis (MBIA) with a body composition analyzer (InBody S10, InBody Co., South Korea) first thing in the morning. Participants were required to fast for at least 12 h overnight and rest in a supine position for at least 15 min after voiding their bladder. Measurements were taken in the supine position using wearable electrodes. The operator placed the right leg electrode between the subject’s right ankle bone and heel, and the left leg electrode in the same manner; both arms were placed flat at the sides of the body, with the left-hand electrode clamped between the middle finger and thumb, and the right-hand electrode positioned in the same manner. The subject remained still and quiet, and the measurement lasted approximately 1–2 min. The instrument automatically performs the analysis using weak, multi-frequency currents. All procedures are carried out by personnel who have undergone standardized training to ensure the standardization and reproducibility of the measurement process.

### 2.4 Equations for predicting resting energy expenditure

The 11 prediction equations used in this study are: (1) Harris-Benedict (15), (2) FAO/WHO/UNU (16), (3) Schofield (17), (4) Fredrix (18), (5) Owen (19), (6) Ganpule (20), (7) Mifflin-St. Jeor (21), (8) Shizgal-Rosa (22), (9) Katch-McArdle (23), (10) Cunningham (24), (11) Thumb (25). The characteristics of each equation are shown in Table 1.

**Table 1:** Characteristics of equations for the prediction of REE.

Equations	Sex	Equation
Harris-Benedict	Male	66.4730 + 13.7516 × W(kg) + 5.0033 × H(cm)-6.7550 × A(y)
	Female	655.0955 + 9.5634 × W(kg) + 1.8496 × H(cm)-4.6756 × A(y)
FAO/WHO/UNU	Male	[36.8 × W(kg) + 4719.5 × (H(cm)/100)-4481]/4.186
	Female	[38.5 × W(kg) + 2665.2 × (H(cm)/100)-1264]/4.186
Schofield	Male	(0.049 × W(kg) + 2.459) × 1000/4.186
	Female	(0.038 × W(kg) + 2.755) × 1000/4.186
Fredrix	Male	1,641 + 10.7 × W(kg)-9.0 × A(y)-203 × 1
	Female	1,641 + 10.7 × W(kg)-9.0 × A(y)-203 × 2
Owen	Male	879 + 10.2 × W(kg)
	Female	795 + 7.18 × W(kg)
Ganpule	Male	(0.0481 × W(kg) + 0.0234 × H(cm)-0.0138 × A(y)-0.4235) × 1000/4.186
	Female	(0.0481 × W(kg) + 0.0234 × H(cm)-0.0138 × A(y)-0.9708) × 1000/4.186
Mifflin-St. Jeor	Male	(10 × W(kg) + 6.25 × H(cm)-5 × A(y)) + 5
	Female	(10 × W(kg) + 6.25 × H(cm)-5 × A(y))-161
Shizgal-Rosa	Male	80.362 + 13.397 × W(kg) + 4.799 × H(cm)-5.677 × A(y)
	Female	447.593 + 9.247 × W(kg) + 3.098 × H(cm)-4.330 × A(y)
Katch-McArdle		370 + 21.6 × FFM(kg)
Cunningham		500 + 22.0 × FFM(kg)
Thumb		W(kg) × 20

W, weight; H, height; A, age; FFM, fat-free mass. For the Fredrix equation, 1 = male and 2 = female.

### 2.5 Subgroup analysis

In terms of demographics, participants were divided into two groups based on gender: male and female. Age groups were defined according to the Chinese standard “Recommended Body Mass Index Ranges and Weight Management Standards for the Elderly” (WS/T 868—2025) (26), with participants categorized into the 60–80 years group and the 80–100 years group. BMI groups were classified according to the same standard, with participants divided into the ≤27.0 kg/m^2^ group and the >27.0 kg/m^2^ group. Regarding lifestyle, daily outdoor activity time was categorized into <1 h and ≥1 h groups using 1 h/day as the cutoff point, in accordance with the Chinese “Risk Assessment of Malnutrition in the Elderly” (WS/T 552—2017) (27); nightly sleep duration was categorized into <5 h and ≥5 h groups using 5 h/day as the cutoff point, in accordance with the same standard; Regarding nutritional status, a two-stage assessment method was adopted based on the Chinese “Risk Assessment of Malnutrition in the Elderly” (WS/T 552—2017) (27): First, a preliminary screening was conducted; a score <12 indicated a risk of malnutrition. If the preliminary score was ≥12, the assessment section was completed, and the total score (combining preliminary and assessment scores) was calculated; a total score <24 also indicated a risk of malnutrition. For muscle assessment, SMI was determined according to the 2019 Consensus of the Asian Sarcopenia Working Group (28). Men with an SMI < 7.0 kg/m^2^ and women with an SMI < 5.7 kg/m^2^ were classified into the low muscle mass subgroup; men with an SMI ≥ 7.0 kg/m^2^ and women with an SMI ≥ 5.7 kg/m^2^ were classified into the high muscle mass subgroup.; For CC, following the Chinese “Risk Assessment of Malnutrition in the Elderly” (WS/T 552—2017) (27), participants are classified into groups with CC < 31 cm and ≥ 31 cm.

### 2.6 Statistical analysis

Statistical analysis was performed using SPSS 21.0 software. Continuous variables are expressed as mean ± standard deviation, and categorical variables are expressed as proportion or percentage. The comparison between measured REE and predicted REE calculated by each predictive equation was conducted as follows: when the difference followed a normal distribution, a paired t-test was used; when the difference did not follow a normal distribution, the Wilcoxon signed-rank test was used; and for comparisons between groups of categorical variables, the chi-square test was used. The following metrics were used for the comprehensive evaluation of the predictive equation’s performance: Bias = (REE_measured_−REE_predicted_)/REE_measured_ × 100,used to reflect the systematic error of the predictive equation (4); Precision = SD (∣REE_measured_−REE_predicted_∣),used to reflect the degree of dispersion of prediction errors (29); Accuracy: the proportion of subjects whose predicted values fall within 10% of the measured values, used to evaluate prediction accuracy at the individual level (30, 31). A Bland–Altman analysis was used to assess the agreement between predicted REE and measured REE; scatter plots were plotted, 95% limits of agreement were calculated, and regression analysis was performed to test for proportional bias (32). All statistical tests were two-sided, and a *p* < 0.05 was considered statistically significant. Additionally, to explore possible nonlinear relationships between continuous variables and REE, a restricted cubic spline model was constructed for further validation.

## 3 Results

### 3.1 Quality control

Weekly ethanol combustion tests demonstrated satisfactory instrument performance throughout the study period. The mean relative errors between the measured and stoichiometrically predicted values were 1.4% for VO₂ and 2.3% for VCO₂, respectively,supporting the validity of the indirect calorimetry measurements. Three independent performance verification sessions were conducted using different subsets of randomly selected participants, collectively accounting for 20% of the original study sample. The mean relative error between the repeat and original REE measurements was 1.9%, with a range of 0.8%–3.1% The mean within-subject coefficient of variation derived from repeated REE measurements was 1.6%, and all relative errors were below the prespecified acceptable limit of 5%, supporting reproducibility of the indirect calorimetry measurements.

### 3.2 Baseline characteristics of participants

This study included a total of 156 elderly residents in nursing homes, with older women accounting for a high proportion of 62.2%. The mean age of the participants was 83.4 ± 5.6 years, and 12.18% of the participants were over 90 years old. 45.5% of the participants were at risk of malnutrition, with a higher prevalence among women than men. REE in the elderly participants was measured using indirect calorimetry, with an overall mean of 1378.6 ± 268.0 kcal/day; the mean for men was 1498.2 ± 263.4 kcal/day, and for women, 1295.5 ± 235.7 kcal/day, with men having a higher REE than women. The baseline characteristics of the study participants are summarized in Table 2.

**Table 2:** Baseline characteristics of the study participants.

Characteristics	Cross-sectional datasets		
	Overall (*N* = 156)	Men (*N* = 59)	Women (*N* = 97)
Age (y)	83.4 ± 5.6	84.3 ± 6.2	82.9 ± 5.2
Age>90y (%)	12.18%	18.64%	8.25%
Weight (kg)	61.9 ± 11.0	67.9 ± 12.1	58.3 ± 8.6
BMI (kg/m^2^)	25.1 ± 3.9	24.5 ± 4.1	25.3 ± 3.8
AMC (cm)	25.0 ± 2.5	26.2 ± 2.9	24.3 ± 2.1
CC (cm)	31.9 ± 5.9	33.5 ± 8.7	31.0 ± 3.0
TBW (kg)	28.5 ± 5.8	33.3 ± 6.0	25.6 ± 3.0
Protein (kg)	7.4 ± 1.5	8.7 ± 1.6	6.7 ± 0.8
Minerals (kg)	2.7 ± 0.5	3.1 ± 0.7	2.5 ± 0.3
BFM (kg)	23.4 ± 7.4	22.9 ± 8.8	23.6 ± 6.5
FFM (kg)	38.7 ± 7.8	45.1 ± 8.3	34.8 ± 4.0
FFMI (Kg/m^2^)	15.5 ± 1.9	16.4 ± 2.4	15.0 ± 1.4
Risk of malnutrition (%)	45.5%	42.4%	47.4%
REE (Kcal/d)	1378.6 ± 268.0	1498.2 ± 263.4	1295.5 ± 235.7
RQ	0.89 ± 0.06	0.88 ± 0.06	0.89 ± 0.06

AMC, arm muscle circumference; CC, calf circumference; TBW, total body water; BFM, body fat mass; FFM, fat-free mass; FFMI, fat-free mass index.

### 3.3 Performance evaluation of equations for predicting resting energy expenditure

**Figure 1:**
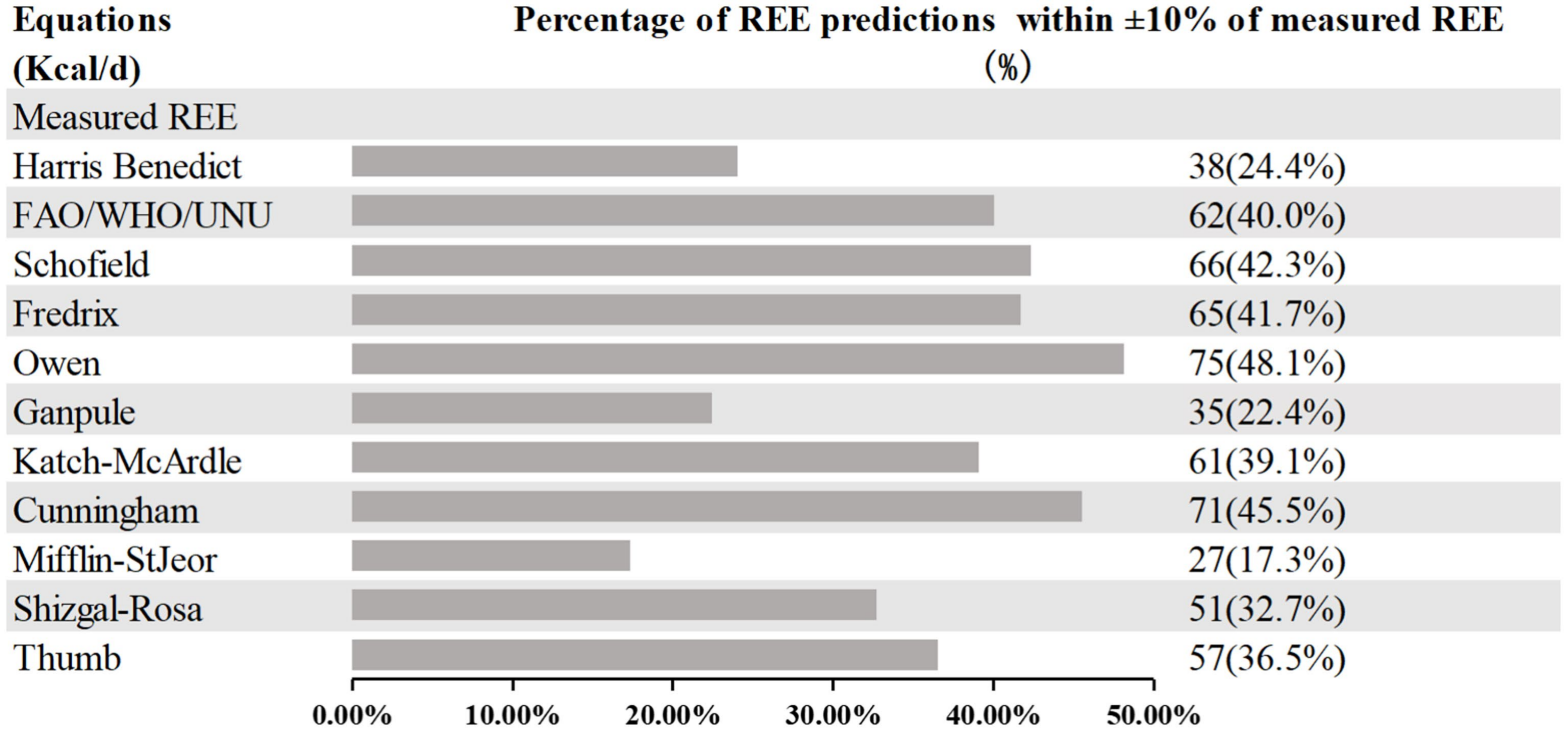
Percentage of REE predictions within 10% of the measured REE for each prediction equation.

**Figure 2:**
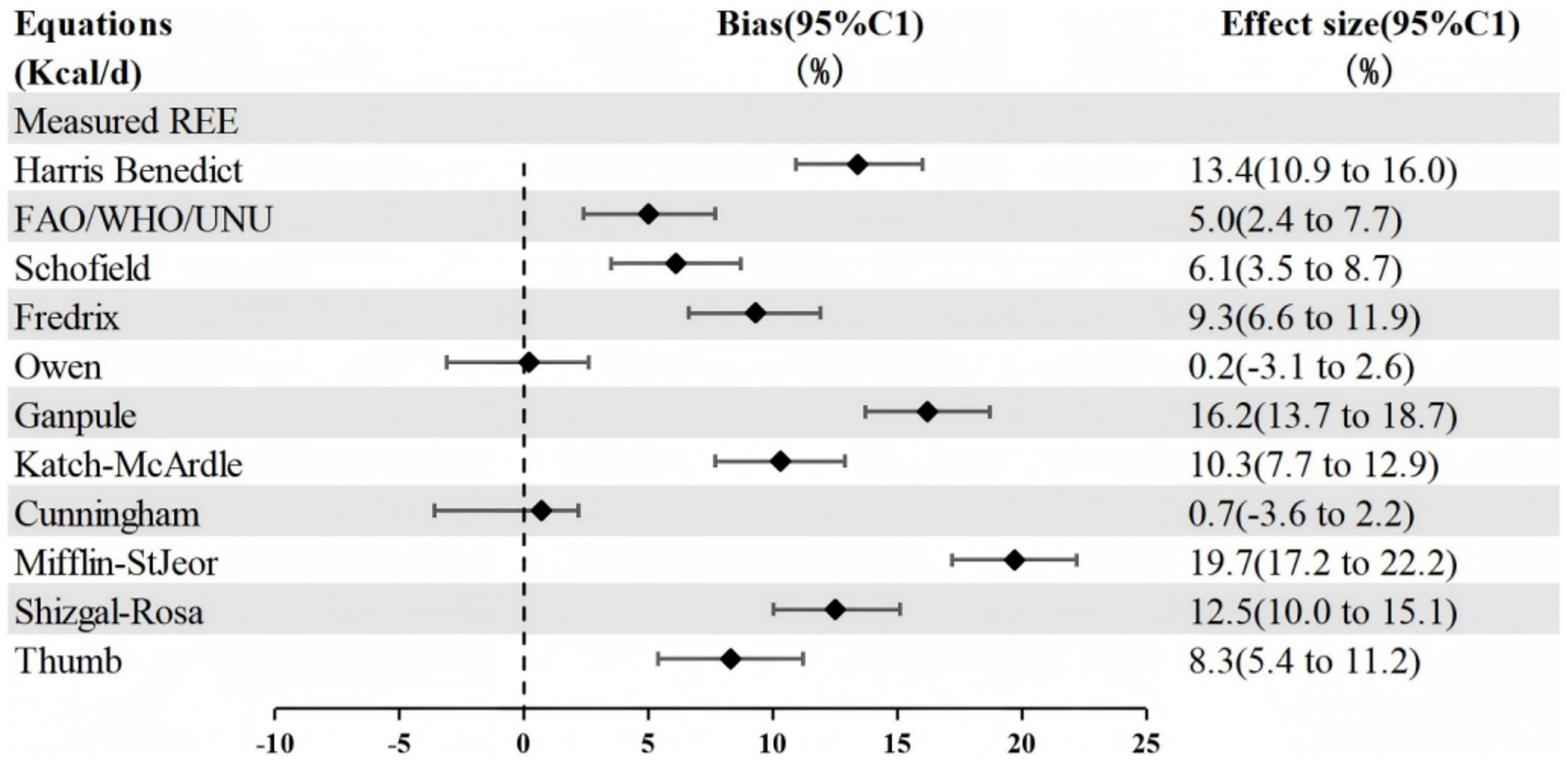
Percent bias of resting energy expenditure (REE) prediction equations compared to the measured REE.

Statistical results indicate that measured REE differs significantly from the estimates provided by most predictive equations. Specifically, with the exception of the Owen equation (*p* = 0.113) and the Cunningham equation (*p* = 0.155), the Harris-Benedict, FAO/WHO/UNU, Schofield, Fredrix, Ganpule, Katch-McArdle, Mifflin-St Jeor, Shizgal-Rosa, and Thumb equations all significantly underestimated REE in elderly residents of nursing homes (*p* < 0.001). To avoid potential Type I errors, this study further applied the Bonferroni correction (*α* = 0.0045) to the paired comparisons between the 11 predictive equations and the measured REE values; the results showed that the statistical conclusions were consistent before and after the correction. Regarding prediction accuracy at the individual level, measured by the proportion of predicted REE values falling within 10% of the measured REE, the results showed significant differences in performance among the equations: the Owen equation had the highest accuracy, at 48.1%; followed by the Cunningham equation at 45.5%; whereas the Mifflin–St Jeor, Ganpule, and HB equations had lower accuracy rates of 17.3%, 22.4%, and 24%, respectively (Figure 1). These results indicate that the Owen and Cunningham equations showed relatively better individual-level predictive performance than the other equations. Regarding prediction accuracy, the dispersion of prediction errors was quantified using standard deviation, and significant differences in dispersion were observed among the equations: the standard deviations (SD) for the Owen and Cunningham equations were lower than those of the other equations, at 150.7 kcal/day and 153.7 kcal/day, respectively. The bias further reveals systematic errors: the mean bias of the Owen and Cunningham equations was lower than that of the other equations, at 0.2 and 0.7%, respectively (Figure 2). Specific performance metrics for each predictive equation are detailed in Table 3.

**Table 3:** Performance comparison of different REE prediction equations in the study participants.

Equations	Mean (95% CI) (Kcal)	*p*-value	Within 10%of mREE (N = 156)	SD (Kcal/day)	Bias^1^ (%)
Measured REE	1378.6 (1336.2, 1421.0)				
Harris Benedict	1164.1 (1109.3, 1189.2)	<0.001	38(24.4%)	188.4	13.4
FAO/WHO/UNU	1274.9 (1251.2, 1298.5)	<0.001	62(40.0%)	162.1	5.0
Schofield	1261.0 (1238.5, 1283.4)	<0.001	66(42.3%)	162.2	6.1
Fredrix	1223.7 (1193.6, 1253.8)	<0.001	65(41.7%)	169.6	9.3
Owen	1348.9 (1317.9, 1379.8)	0.113	75(48.1%)	150.7	0.2
Ganpule	1133.1 (1101.2, 1165.0)	<0.001	35(22.4%)	182.1	16.2
Katch-McArdle	1206.0 (1179.4, 1232.6)	<0.001	61(39.1%)	185.0	10.3
Cunningham	1351.5 (1324.4, 1378.6)	0.155	71(45.5%)	153.7	0.7
Mifflin-StJeor	1086.8 (1054.1, 1119.5)	<0.001	27(17.3%)	171.9	19.7
Shizgal-Rosa	1177.3 (1150.6, 1204.1)	<0.001	51(32.7%)	190.0	12.5
Thumb	1238.5 (1203.6, 1273.4)	<0.001	57(36.5%)	178.8	8.3

CI, confidence interval; SD, standard deviation.

1 Bias (%), calculate as the mean of individual percentage errors: (measured REE−predicted REE)/measured REE×100%.

**Figure 3:**
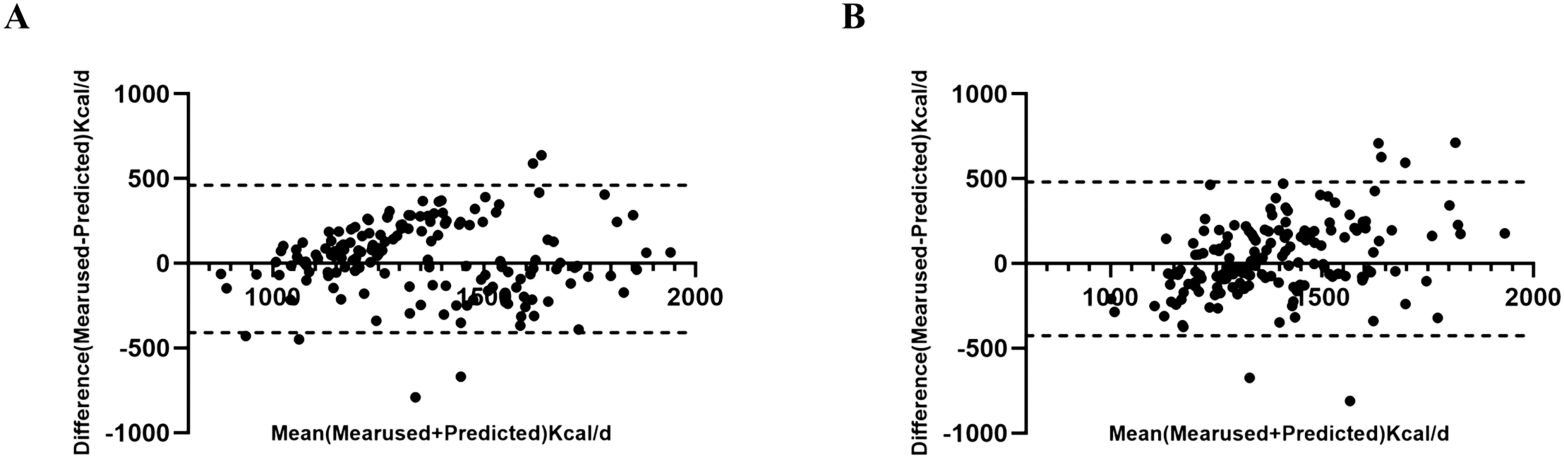
Bland–Altman analysis. **(A)** Bland–Altman plot comparing predicted and measured REE using the Owen equation. **(B)** Bland–Altman plot comparing predicted and measured REE using the Cunningham equation.

Therefore, the Owen and Cunningham equations were selected for further agreement and subgroup analyses because they demonstrated the most favorable overall performance among the evaluated equations. To further evaluate the agreement between the predicted REE from these two equations and the measured REE, this study employed the Bland–Altman analysis method. The results show that for the Owen equation, the 95% limits of agreement (95% LoA) range from −401.2 to 460.6 kcal/d, The results of the proportional bias test showed that there was no significant linear relationship between the difference and the mean (Slope = 0.10, *p* = 0.89). For the Cunningham equation, the 95% LoA for the difference between predicted and measured REE were −419.7 to 473.9 kcal/d; The results of the proportional bias test showed that no significant linear relationship was observed between the difference and the mean (Slope = 0.21, *p* = 0.12) (Figure 3).

The above results indicate that the Owen and Cunningham equations showed relatively better agreement with measured REE than the other equations evaluated. However, the 95% LoA for both equations are relatively wide, suggesting that significant differences may still exist between predicted and measured REE at the individual level. In summary, compared with traditional equations such as the Harris-Benedict, FAO/WHO/UNU, and Schofield equations, among the 11 equations evaluated, the Owen and Cunningham equations demonstrated the most favorable overall performance among the evaluated equations in the elderly population residing in nursing homes.

### 3.4 Subgroup analysis

An examination of the above results reveals that even the Owen and Cunningham equations—which demonstrated the best predictive performance among all equations—had accuracy rates that did not exceed 50%. To further investigate the factors influencing the predictive performance of these two equations, this study conducted subgroup analyses based on variables such as gender, age, BMI, SMI, CC, sleep duration, time spent outdoors, and nutritional status. Table 4 presents a comparison of the predictive performance of the Owen and Cunningham equations across different subgroups. The results indicate significant differences in predictive accuracy between the two equations in certain subgroups. In the age subgroup analysis, the Owen equation had a prediction accuracy of only 24.1% for the 60–80 years group, whereas its accuracy significantly improved to 53.5% in the 80–100 years group, with a statistically significant difference in accuracy between the two groups (*p* < 0.01); In contrast, the difference in accuracy between these two age subgroups for the Cunningham equation was not statistically significant (*p* = 0.247). In the subgroup analysis based on muscle mass, the Cunningham equation showed significantly higher accuracy in predicting REE in older adults with high muscle mass than in those with low muscle mass (*p* < 0.05), whereas the Owen equation showed significantly higher accuracy in predicting REE in older adults with low muscle mass than in those with high muscle mass (*p* < 0.05). In the nutritional status subgroup analysis, the Cunningham equation showed significantly higher accuracy in predicting REE for older adults without a risk of malnutrition compared to those at risk of malnutrition (*p* < 0.05), whereas the difference in accuracy between the two groups using the Owen equation was not statistically significant (*p* = 0.781). To assess the reliability of significant results and the reliability of significant subgroup results, this study conducted a Post-hoc statistical power analysis. The results showed that the statistical power of the Owen equation in the age subgroup analysis reached 97.7% (Power = 0.977), and the statistical power of the Owen equation in the muscle mass subgroup analysis reached 70.0% (Power = 0.700). The Cunningham equation had a statistical power of 70.0% (Power = 0.700) in the muscle mass subgroup analysis, and the Cunningham equation had a statistical power of 86.4% (Power = 0.864) in the malnutrition subgroup analysis, suggesting that the relevant results have moderate to high statistical power.

**Table 4:** Subgroup analysis of REE prediction differences: Owen and Cunningham equations.

Subgroup	Owen				Cunningham			
	SD (Kcal/day)	Bias^2^ (%)	Within 10% of mREE (*N* = 156)	*p*-value^1^	SD (Kcal/day)	Bias^2^ (%)	Within 10% of mREE (*N* = 156)	*p*-value^1^
Sex				0.834				0.704
Men	161.4	−7.6	29 (49.2%)		186.2	−0.2	28 (47.4%)	
Women	122.2	3.5	46 (47.4%)		145.8	0.4	43 (44.3%)	
BMI (kg/m^2^)				0.199				0.865
≤27	126.9	0.5	57 (51.4%)		134.8	−1.2	51 (45.9%)	
>27	173.8	−1.0	18 (40.0%)		190.2	1.0	20 (44.4%)	
Age (year)				<0.01				0.247
60–80	189.3	3.3	7 (24.1%)		141.5	0.6	16 (55.2%)	
80–100	137.2	−1.0	68 (53.5%)		186.4	−0.9	55 (43.3%)	
SMI (kg/m^2^)				<0.05				<0.05
Low muscle mass	122.3	−2.1	21 (63.6%)		239.7	−14.0	10 (30.3%)	
High muscle mass	152.1	0.6	54 (43.9%)		120.7	10.5	61 (49.6%)	
CC (cm)				0.302				0.231
<31	126.6	−2.2	31 (53.4%)		121.5	−2.9	30 (51.7%)	
≥31	160.2	1.0	44 (44.9%)		163.7	0.6	41 (41.8%)	
Sleep time (h/d)				0.160				0.506
<5	171.3	−3.0	12 (63.2%)		149.3	−3.5	10 (52.6%)	
≥5	154.8	−0.4	63 (46.0%)		157.2	−0.8	61 (44.5%)	
Outdoor time (h/d)				0.688				0.967
<1	144.9	−3.7	32 (50.0%)		160.6	−2.0	29 (45.3%)	
≥1	154.2	2.2	43 (46.7%)		148.6	0.2	42 (45.7%)	
Malnutrition				0.781				<0.05
Yes	164.7	−1.2	35 (49.3%)		167.3	−1.8	23 (32.4%)	
No	140.2	−0.3	40 (47.1%)		133.4	0.2	48 (56.5%)	

SD, standard deviation.

1 Pearson’s chi-square test was used for comparisons of categorical variables.

2 Bias (%), calculate as the mean of individual percentage errors: (measured REE−predicted REE)/measured REE×100%.

In terms of precision and bias, the Owen equation performed better in the 80–100 years subgroup than in the 60–80 years subgroup: the standard deviation of the Owen equation in the 60–80 years subgroup was 189.3 kcal/day, with a mean bias of 3.3%; whereas in the 80–100 years subgroup, the standard deviation was 137.2 kcal/day and the mean bias was −1.0%. Similarly, the Cunningham equation demonstrated greater accuracy and less bias in the subgroup of older adults with high muscle mass and no risk of malnutrition compared to the comparison subgroup.

In summary, factors closely related to metabolic characteristics—such as age, muscle mass, and malnutrition—exhibited significant and oppositely directed associations with the accuracy, precision, and mean bias of the Owen and Cunningham equations, suggesting that these characteristics may be key variables influencing the applicability of the two equations for predicting REE in older adults residing in long-term care facilities.

### 3.5 Restricted cubic spline model fitting

**Figure 4:**
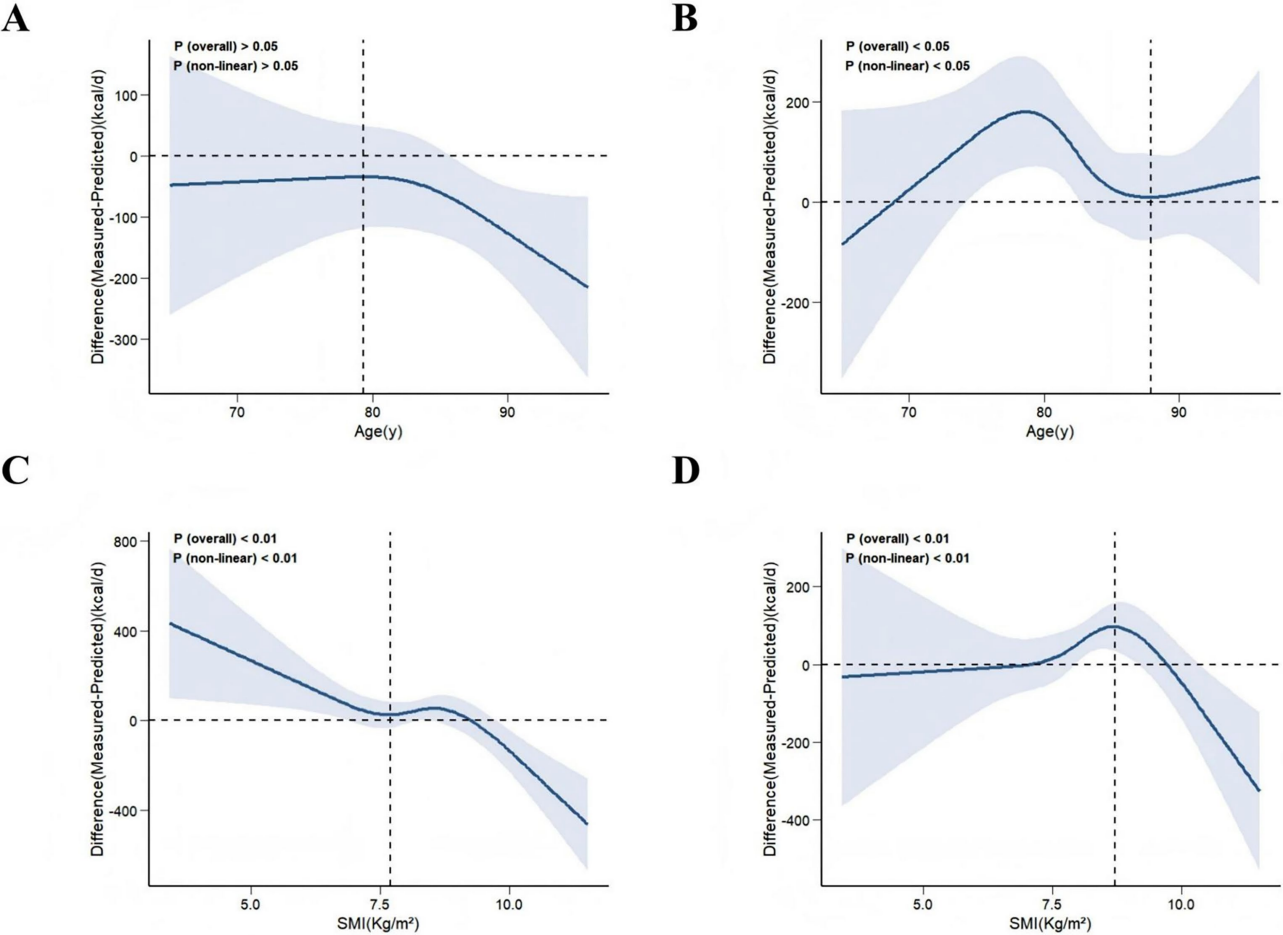
Restricted cubic spline analysis. **(A)** The association between age and the difference between measured REE and Cunningham-predicted REE. **(B)** The association between age and the difference between measured REE and Owen-predicted REE. **(C)** The association between SMI and the difference between measured REE and Cunningham-predicted REE. **(D)** The association between SMI and the difference between measured REE and Owen-predicted REE.

To further explore the factors affecting the performance of the prediction equation, this study constructed a restricted cubic spline (RCS) model, with prediction error (measured REE – predicted REE) as the dependent variable and age and SMI as independent variables, respectively. The model was fitted using four knots set at the 5th, 35th, 65th, and 95th percentiles of the independent variable distributions, with the median serving as the reference value. The model also incorporated variables such as gender and calf circumference (CC) as covariates for adjustment. The results of the RCS model show that the prediction error of the Cunningham Equation did not exhibit a nonlinear trend with increasing age (Figure 4A), and the nonlinear relationship was not statistically significant (*P*(overall) > 0.05, P(non-linear) > 0.05). In contrast, the prediction error of the Owen Equation exhibited a typical nonlinear trend with increasing age (Figure 4B)—in the 60–80 years group, the prediction error of the Owen Equation values rose significantly and exhibited a wider range of variation; whereas in the 80–100 years group, the prediction error of the Owen Equation values declined rapidly and approached 0. This nonlinear relationship was statistically significant (*P*(overall) < 0.05, P(non-linear) < 0.05). Similarly, the prediction error of the Owen equation exhibits a typical nonlinear trend with increasing SMI (Figure 4D): it approaches 0 within the range where SMI is below approximately 7.0 kg/m^2^, whereas in the range where SMI exceeds approximately 7.0 kg/m^2^, the prediction error of the Owen Equation first increases and then decreases. The nonlinear association is statistically significant (*P*(overall) < 0.01, *P*(non-linear) < 0.01). These results indicate that the Owen equation demonstrates superior predictive performance for older adults aged 80–100 in long-term care facilities and for older adults with low muscle mass, which is highly consistent with the results of the subgroup analysis. In contrast, the prediction error of the Cunningham equation exhibits a typical nonlinear trend as SMI increases (Figure 4C). Within the range where SMI is below approximately 7.0 kg/m^2^, the prediction error of the Cunningham equation is positive, suggesting that the measured values are significantly higher than the predicted values. It then gradually approaches zero, with the prediction error of the Cunningham equation being closest to 0 near an SMI of approximately 7.5 kg/m^2^. When SMI exceeds approximately 9.0 kg/m^2^, the prediction error of the Cunningham equation turns negative and continues to decrease. Overall, a significant nonlinear relationship is observed (*P*(overall) < 0.01, *P*(non-linear) < 0.01). This suggests that the Cunningham equation tends to underestimate REE in older adults with low muscle mass living in nursing homes, whereas it demonstrates good predictive accuracy in individuals with high muscle mass. Observation of the 95% confidence interval width of the RCS curve reveals that the Cunningham equation has a relatively narrow confidence interval when SMI exceeds approximately 7.0 kg/m^2^, suggesting lower prediction variability and more stable results in older adults with better muscle mass.

The results of the RCS model clearly indicate that the Owen equation demonstrates superior predictive performance among elderly residents of nursing homes aged 80–100 and patients with low muscle mass, whereas the Cunningham equation performs better among elderly residents of nursing homes with higher muscle mass; these findings are consistent with the results of the subgroup analysis.

## 4 Discussion

This study systematically evaluated the applicability of 11 commonly used resting energy expenditure (REE) prediction equations among older adults in long-term care facilities. Based on a comprehensive analysis of bias, precision, accuracy, and consistency, the Owen and Cunningham equations performed better overall than the other prediction equations. Both exhibited low systematic error and relatively better predictive precision, and also demonstrated higher predictive accuracy at the individual level compared to the other equations. Specifically, these two equations exhibited the highest accuracy (48.1 and 45.5%, respectively), the highest precision (with standard deviations of 150.7 kcal/day and 153.7 kcal/day, respectively), and the smallest bias (0.2% and 0.7%, respectively). Further Bland–Altman analysis indicated that the Owen and Cunningham equations showed relatively better agreement with measured REE than the other equations evaluated. These results are similar to those reported by Griffith et al. (33) in their study of obese older adults. Griffith found that the Owen equation exhibited the lowest variability and an accuracy rate of 50.7%; the Mifflin equation had the lowest accuracy, with only 43.1% of its predicted values deviating by less than 10% from the gold standard measured by indirect calorimetry, and it also showed greater variability. The advantages of the Owen and Cunningham equations may be attributed to their variable design. For example, the Cunningham equation estimates REE using FFM values; this equation is widely used in the fields of nutrition and fitness and provides a more comprehensive reflection of the impact of factors such as muscle mass and nutritional status on metabolism (24, 34), which is particularly important for older adults with reduced muscle mass. The Owen equation was originally developed using body weight as the sole predictor and may be less affected by age-related changes in body composition measurement. In very old adults, where alterations in hydration status and body composition are common, the simplicity of the Owen equation may partially explain its relatively stable performance. The results of the subgroup analysis in this study indicate that, compared with older adults with normal muscle mass and good nutritional status, the Cunningham equation demonstrated significantly higher accuracy in predicting their metabolic status. A study by Lahaye et al. (10) on residents of nursing homes for the elderly similarly pointed out that prediction errors in traditional equations such as those from the WHO and HB are influenced by muscle mass, and that FFM and CRP levels are also key determinants of REE. This further indicates that the basal metabolic rate in older adults is influenced by multiple variables related to physical aging and cannot be explained solely by changes in variables such as height and age.

It is worth noting in the accuracy evaluation that the 10% accuracy standard is a widely adopted evaluation metric in validation studies of REE prediction equations (30, 31). In the study population, this error range is approximately equivalent to 130–150 kcal/day. Given that long-term cumulative errors in energy estimation may affect the effectiveness of nutritional management in older adults, this finding holds clinical significance. However, when evaluated based on the criterion that predicted REE must fall within 10% of measured REE, neither the Owen equation nor the Cunningham equation achieved an accuracy rate exceeding 50%. These results indicate that even the prediction equations that performed relatively best in this study struggle to fully account for the substantial individual metabolic variations among older adults in long-term care facilities. This suggests that existing equations have limitations when applied to older adults in Chinese long-term care facilities, and indirect calorimetry remains the method of choice for measuring REE. This is consistent with the findings of several population-based studies. For example, studies by Reidlinger et al. (9), Siervo et al. (35),and others have shown that traditional equations such as the FAO/WHO/UNU, Mifflin, and Harris-Benedict equations have accuracy rates ranging from 40% to 60% in healthy older adults, while their predictive accuracy further decreases in institutionalized older adults with chronic diseases. A study by Kawase et al. (4)on Elderly people in Japanese hospitals also found that most commonly used prediction equations failed to keep the error within 10% for more than 60% of individuals-specifically, the FAO/WHO/UNU and Schofield equations often overestimate REE, while the Harris-Benedict and Ganpule equations tend to underestimate it. The reason for these findings may be that commonly used predictive equations rarely include older adults as the primary subjects in validation studies (35), and most were developed based on North American or European populations-for example, the database for the Schofield equation includes thousands of participants, 45% of whom are of Italian descent, a group with a basal metabolic rate significantly higher than that of other ethnic groups, which may cast doubt on the equation’s predictive performance for older Asian populations (36)Furthermore, REE may decrease with advancing age due to factors such as reduced muscle mass, a decline in basal metabolic rate, and decreased physical activity (35); conversely, REE may increase due to elevated metabolic turnover, fever, or the high metabolic effects of certain medications (4). However, previous studies typically included only conventional variables such as age and height, and did not adequately standardize for age, making it difficult to fully account for age-related changes in body composition. In this specialized study of elderly residents in nursing homes, most predictive equations significantly underestimated REE, which may be closely related to the fact that the aforementioned factors were not adequately accounted for or corrected in traditional predictive equations.

From a clinical perspective, this study recommends that indirect calorimetry be the method of choice for nutritional management in long-term care facilities; if resources are limited, the Owen or Cunningham equations may be used, but they must be adjusted accordingly based on factors such as the patient’s age, nutritional status, and muscle mass. This study contributes to the development of precise energy intake plans However, the following limitations also exist: Firstly, although rigorous quality-control procedures were implemented, including routine pre-measurement calibration, weekly ethanol combustion validation, and post-study performance verification with repeated REE measurements, some degree of measurement error inherent to indirect calorimetry cannot be entirely excluded. Factors such as minor instrument drift, environmental fluctuations, and biological variability among participants may still have influenced REE measurements. Therefore, the findings should be interpreted in light of these potential sources of measurement uncertainty. Secondly, this study conducted multiple comparative analyses and subgroup analyses; therefore, the risk of Type I errors resulting from multiple comparisons cannot be completely ruled out. Although the study conclusions remained stable after applying Bonferroni correction to the analysis of the 11 equations, some subgroup analysis results were not adjusted for multiple comparisons because subgroup analyses were primarily used to explore potential influencing factors. Thirdly, although the Owen and Cunningham equations performed best among those evaluated and no significant proportional bias was observed, their 95% limits of agreement were relatively wide, indicating substantial individual variation between predicted and measured REE. This variation may correspond to clinically significant differences in daily energy prescriptions for frail older adults. Fourthly, although the overall sample size of this study reached 156 participants, the sample sizes for some subgroup analyses were relatively limited, which may affect the stability of effect estimates and the reproducibility of results and these results need to be further validated in future studies with larger samples and independent cohorts. Finally, this study employed a cross-sectional design without longitudinal follow-up; therefore, it was not possible to assess the long-term applicability of the different predictive equations in the context of changes in nutritional status or during nutritional interventions.

Future research should conduct multicenter, longitudinal studies to further externally validate existing predictive equations and, by incorporating anthropometric characteristics, body composition features, and health status specific to Asian older adults, establish REE prediction models tailored to Asian older adults—particularly those in China—to improve the accuracy of individualized nutritional assessments.

## Data Availability

The raw data supporting the conclusions of this article will be made available by the authors, without undue reservation.
