# Supplementary material for: Applicability of resting energy expenditure prediction equations in Chinese nursing home residents
Source: Front Nutr. 2026 Jul 28;13:1860294. doi: 10.3389/fnut.2026.1860294 (PMC13457139; doi:10.3389/fnut.2026.1860294)
Supplement: Supplementary file 1 [file Supplementary_file_1.docx]

**Supplementary Figure S1.** Flow diagram of participant recruitment and selection
